# Health Care Providers’ Perspectives on Early Warning Systems for Acute Respiratory Infections in Canada: Qualitative Study

**DOI:** 10.2196/85244

**Published:** 2026-04-23

**Authors:** Jinfan Qiang, Kevin Maruthananth, Maaha Farrukh, Andrew David Pinto, Banafshe Hosseini

**Affiliations:** 1 Upstream Lab, MAP Centre for Urban Health Solutions, St. Michael's Hospital, 30 Bond Street, Toronto, ON, M5B 1W8, Canada, 1 416-864-6060 ext 76148; 2 Institute of Health Policy, Management, and Evaluation, Dalla Lana School of Public Health, University of Toronto, Toronto, ON, Canada; 3 Department of Family and Community Medicine, St. Michael's Hospital, Unity Health Toronto, Toronto, ON, Canada; 4 Department of Family and Community Medicine, Faculty of Medicine, University of Toronto, Toronto, ON, Canada; 5 Division of Clinical Public Health, Dalla Lana School of Public Health, University of Toronto, Toronto, ON, Canada

**Keywords:** early warning system, pandemic preparedness, qualitative method, primary care, health care

## Abstract

**Background:**

Acute respiratory infections (ARIs) remain a significant global health challenge and are the second leading cause of disease burden and mortality. Early warning systems (EWS) play a key role in detecting clinical deterioration, alerting health care providers (HCPs), and supporting pandemic surveillance. While existing literature highlights HCPs’ positive experiences with EWS in confirming clinical assessments and guiding escalation, perspectives on how these systems can be optimized for ARI management remain underexplored.

**Objective:**

As Canada continues to develop and operationalize EWS for outbreak and pandemic preparedness, this study aims to explore the experiences and insights of primary care providers, emergency department (ED) physicians, and researchers regarding the use of EWS for ARI management in Canada.

**Methods:**

Eleven participants, including primary care providers, ED physicians, and researchers from urban and rural settings across 5 Canadian provinces (Ontario, Newfoundland and Labrador, Quebec, British Columbia, and Manitoba), were recruited in 2024. All participants regularly managed patients with ARIs or played key roles in pandemic response. A codebook thematic analysis was conducted to identify patterns and themes, with subthemes organized under broader thematic categories. Data saturation was assessed during the analysis phase. The study adhered to the COREQ (Consolidated Criteria for Reporting Qualitative Research) guidelines.

**Results:**

Among the 11 participants, there was approximately equal representation across gender and age groups, and more than 90% had over ten years of experience in ARI management. Three overarching themes emerged. First, participants demonstrated general awareness of the use of EWS in ARI management, including outbreak detection, screening and triage support, and informing clinical decision-making. Technologies and surveillance tools used during the COVID-19 pandemic were frequently referenced; however, understanding of specific EWS and their application to ARI management was often limited. Second, participants identified key attributes of an effective EWS as accuracy, timeliness, integration, and equity, emphasizing the need for seamless integration into existing Canadian health care workflows without increasing administrative burden. Third, anticipated challenges were described across 4 stages of EWS development, including initiation (funding and privacy concerns), implementation (outdated data systems and limited legislation), use (staff shortages and capacity constraints), and evaluation (lack of standardized and innovative evaluation approaches).

**Conclusions:**

This study engaged 11 experienced HCPs and researchers who were directly involved in patient care and public health response to ARI outbreaks and qualitatively explored their perspectives on EWS for ARI management and pandemic preparedness. The findings identified 3 overarching themes regarding the general knowledge, desired attributes, and anticipated challenges of EWS in ARI management, highlighting the importance of co-designing EWS with clinicians, researchers, and other key stakeholders to improve their effectiveness and integration into clinical practice and pandemic preparedness across Canada.

## Introduction

Acute respiratory infections (ARIs) affect the upper and/or lower respiratory tract, ranging from mild to life-threatening illnesses, and remain a significant public health concern [1], particularly among children [2]. Over the past 2 decades, severe ARIs, including SARS-CoV-2, have caused significant morbidity and mortality worldwide [3-5]. In 2021, SARS-CoV-2 was the second leading cause of global mortality and the leading cause of disability-adjusted life years [6]. Given the highly contagious nature and rapid transmission of ARIs, early detection and timely intervention are essential to mitigating their impact and preventing further transmission.

Early warning systems (EWS) are designed to identify clinical deterioration, alert health care providers (HCPs), and facilitate timely intervention [7,8]. These systems have been widely implemented in acute care settings such as hospitals and emergency departments (EDs) [9-11] and have also been integrated into outpatient and community settings, including general practices and health centers [12,13]. Primary care and ED settings play complementary roles in early outbreak detection; primary care often serves as the first point of contact for patients with early or nonspecific symptoms, while EDs act as sentinel sites during outbreaks by managing surges of patients with acute presentations. Syndromic surveillance using primary care and ED data has been shown to be a valuable tool for early outbreak detection during the COVID-19 pandemic [14]. However, both settings face distinct implementation challenges, including data interoperability and real-time integration in primary care and crowding, time pressure, and workforce constraints in EDs [15,16]. These contextual and workflow-related factors influence how EWS are used in practice, underscoring the importance of understanding frontline clinicians’ perspectives when considering EWS implementation and optimization.

In Canada, multiple surveillance and EWS platforms monitor ARIs at various levels of the health care system. FluWatchers, a voluntary, self-reported system operated by the Public Health Agency of Canada, collects data on flu-like symptoms to track respiratory virus activity across the country [17]. The TARRANT Viral Watch initiative links primary care clinics and laboratories to monitor ARI trends in real time [18]. The Sentinel Practitioner Surveillance Network (SPSN) engages health care providers in tracking clinical and laboratory data to assess the epidemiology of influenza and other respiratory pathogens [19]. The Serious Outcomes Surveillance (CIRN SOS) network operates within hospitals to monitor severe ARI cases and evaluate disease burden and vaccine effectiveness [20]. However, challenges related to data interoperability and fragmented governance across jurisdictions remain.

Internationally, EWS-related approaches for ARI include both clinical risk stratification tools and population-based surveillance systems. For example, the National Early Warning Score (NEWS and NEWS2) is used in the United Kingdom, and it has been evaluated during COVID-19 for predicting mortality and Intensive Care Unit admission [21]. In contrast, population-based hospitalization surveillance networks such as the Respiratory Virus Hospitalization Surveillance Network (RESP-NET) in the United States monitor laboratory-confirmed hospitalizations for major respiratory viruses to support situational awareness and public health response [22].

While these systems provide valuable surveillance data, they are not consistently integrated into real-time clinical decision-making, and frontline patient management remains limited. As a result, little research examined how EWS are experienced and used within primary care and ED settings in Canada [23,24] or how system-level limitations affect their practical operations [25]. Understanding these perspectives is crucial for informing optimal EWS implementation and effectiveness in ARI management and pandemic preparedness. Accordingly, this study aims to explore the experiences and insights of primary care providers, ED physicians, and researchers regarding the use of EWS for ARI management in Canada.

## Methods

### Overview

This study was conducted following the COREQ (Consolidated Criteria for Reporting Qualitative Research (Checklist 1) [26].

### Participant Recruitment

Individual online interviews were conducted with health care providers and researchers involved in pandemic response using the Zoom video platform (Zoom Video Communications). Eligible participants included family physicians, ED physicians, and infectious disease specialists whose work primarily involves managing ARIs. Researchers with expertise in pandemic response were also included. Inclusive eligibility criteria ensured representation across a range of clinical roles and practice settings, including providers from both urban and rural contexts. This approach enabled the capture of diverse perspectives from professionals directly impacted by EWS and supported a comprehensive understanding of potential challenges and needs in ARI surveillance. Participants were recruited through professional clinical and research networks focused on primary care, emergency medicine, infectious diseases, and pandemic preparedness, including investigator networks associated with adaptive platform trials, respiratory infection surveillance initiatives, and health services research collaborations in Canada. Recruitment was conducted via email using a standardized recruitment script approved by the Unity Health Toronto Communications Department. Recruitment took place between August and October 2024. The response rate was approximately 52%, with eleven of twenty-one invited individuals participating in the study.

### Data Collection

Interviews were conducted by 2 researchers (KM and MF) between August and October 2024. At the beginning of each session, participants completed a brief demographic questionnaire consisting of 9 questions. The demographic questionnaire collected information on participants’ professional role, years of relevant experience, practice setting (eg, urban or rural), and involvement in ARI management or pandemic response. Responses were manually recorded to ensure confidentiality, and this portion was not audio-recorded. A semistructured interview guide was informed by relevant literature on EWS and ARI surveillance, as well as the study objectives and the research team’s research experience (see Multimedia Appendix 1). The guide was designed using Bearman’s step-wise approach [27] to interview schedule development and followed DeJonckheere and Vaughn’s framework [28] for semistructured interviewing in primary care research. The interview guide was not formally pilot tested; however, it was reviewed and refined by the research team prior to data collection and iteratively adapted during the study to reflect participants’ experiences and explore perspectives on the design, implementation, and evaluation of EWS. The full semistructured interview guide, including all questions, is provided in Multimedia Appendix 1.

### Data Analysis

All interviews were audio-recorded, transcribed, and deidentified by a professional transcription service. Participants were offered the opportunity to review their transcripts for accuracy; however, no participants requested transcript review or provided feedback.

Data were analyzed using a codebook-informed reflexive thematic analysis, following Braun and Clarke’s 6-phase framework [29-31]. An inductive approach was used to allow themes to emerge directly from the data. All transcripts were coded and analyzed by a single researcher (JQ), who has training and prior experience in qualitative methodology. Iterative feedback from the principal investigator (BH) was used to refine the coding framework and interpretation. Following familiarization with the data, an initial set of codes was generated and iteratively refined as analysis progressed [32,33]. A finalized codebook was developed and validated by applying it to a new transcript before proceeding with full coding. A hierarchical coding structure outlining themes, subthemes, and example codes is provided in Multimedia Appendix 2.

A codebook was developed to support analytic organization and was refined through iterative application across transcripts. Subthemes were developed when related codes clustered under a broader theme. Reflexivity was maintained throughout the analysis, with process through memoing and regular discussions within the research team, which were used to document reflections, potential biases, interpretive decisions, and support analytic rigor [34].

Interviews were coded using NVivo software (version 14 plus; Lumivero). Data analysis began after all interviews were completed. Data saturation was assessed through ongoing discussions, where it was determined that additional data collection was unlikely to yield new insights [35]. Regular meetings were held with the study team to ensure alignment in interpretation and contextual accuracy.

### Ethical Consideration

This study was approved by the Unity Health Toronto Research Ethics Board (REB 24‐101) prior to the initiation of data collection. The informed consent process, including the consent form, was reviewed and approved by the REB. Written informed consent was obtained via email from all participants before participation. Participants were informed that their participation was voluntary and that they could withdraw from the study at any time without consequence.

To protect participant privacy and confidentiality, interviews were transcribed and deidentified by a professional transcription service before being shared with the research team. All study materials and data were securely stored on a restricted-access drive on St. Michael’s Hospital’s server and were accessible only to research staff approved under the REB application. Interviews were scheduled for one hour, and participants received a CAD $40 (approximately US $ 30, based on an exchange rate of 1 CAD ≈ US $ 0.74 in 2024) honorarium as compensation for their time.

## Results

### Participant Characteristics

Eleven participants from across Canada (Ontario, Newfoundland and Labrador, Quebec, British Columbia, and Manitoba) were interviewed between August and October 2024, with most interviews lasting 30-45 minutes. Demographic characteristics are summarized in Table 1. Participants were evenly distributed by sex (6/11, 55% male) and age group (6/11, 55% aged 30‐44 years). The most common occupation was family physician (5/11, 45%), followed by ED physicians (2/11, 18%), infectious disease physicians (2/11, 18%), a critical care physician (1/11, 9%), and a primary care scientist (1/11, 9%). Most (10/11, 90%) had over 10 years of experience, and 9 out of 11 (82%) worked in urban settings. Regarding EWS knowledge, most participants reported moderate familiarity, while 27% (3/11) had minimal knowledge, and 9% (1/11) were highly knowledgeable.

**Table 1.** Demographic characteristics of participants in a qualitative interview study examining early warning systems (EWS) for acute respiratory infection management among health care providers and researchers in Canada, 2024 (n=11).

Participant characteristics	Values, n (%)
Sex	
Male	6 (55)
Female	5 (45)
Age range (years)	
30‐44	6 (55)
45‐59	5 (45)
Occupation	
Family physician	5 (45)
Emergency department physician	2 (18)
Others^a^	4 (36)
Years of experience (years)	
≤10	1 (9)
11‐15	6 (55)
16‐19	2 (18)
≥20	2 (18)
Geographic Setting	
Urban	9 (82)
Rural	2 (18)
Experience with EWS^b^	
Minimal	3 (27)
Moderate	7 (64)
High	1 (9)

a Others: critical care physician, infectious disease and critical care physician specialist, infectious disease physician, and scientist.

b EWS: early warning system.

### Identified Themes

Three main themes were identified from the analysis: (1) general knowledge of EWS for ARI, (2) desired attributes of EWS for ARI, and (3) anticipated challenges in EWS implementation. Within each theme, 3-4 subthemes were identified.

### Theme 1: General Knowledge of EWS for ARI

Participants reported varying levels of experience with EWS but demonstrated a broad understanding of their role in ARI outbreak monitoring and detection. Most discussions focused on lessons learned from the COVID-19 pandemic, with specific emphasis on the utility of EWS in ARI detection and management.

#### General Awareness

While all participants were familiar with the general concept of EWS, many struggled to name specific systems used for ARI in Canada. When asked for examples, some participants mentioned “wastewater surveillance” (n=3) or “nasopharyngeal swab testing” (n=2), often describing these as ways to track infection trends at the population level rather than as formal EWS. Others referred to public health outbreak updates or case reporting systems as early warning tools. EWS was often described as “a vague term” (P02, ED physician) or “an abstract concept, something of the future” (P05, primary care scientist). Overall, participants’ understanding of EWS appeared to be shaped by their experiences during the COVID-19 pandemic, with limited distinction made between diagnostic testing, population-level surveillance, and structured EWS. One family physician reflected on their limited familiarity,

*I might have to downgrade my other response… not as knowledgeable as I thought I was in terms of specific early warning systems.*

[P08, Family physician]

There was a perceived lack of well-developed national surveillance systems, with some participants stating that provincial-level wastewater surveillance was the only system they had direct experience with. One participant explained,

*National surveillance is not really yet fully developed… at the provincial level, we have wastewater surveillance, but we don’t have experience with a better EWS.*

[P01, Infectious disease physician]

A participant with public health training described the disconnect between EWS and clinical practice, stating,

*I have training in public health… worked in low-income settings and infectious disease control … so I have some concept of what they are. If this question is about how it’s relevant to my current practice, I don’t think I truly understand how the early warning or early detection systems work and maybe that’s because they don’t work or don’t exist in our current clinical environment.*

[P07, ED physician]

Participants’ self-reported familiarity with early warning systems was collected descriptively and was not a primary analytic focus.

#### Insights and Tools Learned From the COVID-19 Pandemic

Participants referenced machine learning, artificial intelligence, and electronic communication as tools that could be integrated into EWS for ARI, although discussions on these technologies were brief. COVID-19 was frequently mentioned as a turning point in EWS discussions, highlighting both challenges and innovations. The complexity of SARS-CoV-2, particularly its “asymptomatic transmission” and *“*atypical symptoms,” was noted as an obstacle to outbreak surveillance and management (P02, ED physician). However, the pandemic also led to rapid adoption of new technologies and scaling of public health interventions.

*The wastewater surveillance is … a novel approach. We did not do this prior to COVID-19, at least in a widespread way. So, the lessons learned were adopt new technology, do it quickly and we can scale it up during the high-pressure pandemic situation.*

[P01, Infectious disease physician]

Four participants also indicated that public health agencies played a crucial role in providing outbreak updates and disseminating information during COVID-19. One participant noted,

*We would rely on public health to tell us … there’s an outbreak… then we… institute… more formal screening process at triage so that we are isolating people quickly…It would be public health looking at the collective data… to let us know that they’re starting to see something new come up and to be on the lookout.*

[P07, ED physician]

#### Role of EWS in ARI Management

Participants identified 3 key functions of EWS in ARI control and management:

First, most participants highlighted the role of EWS in detecting early signals of outbreaks, particularly by identifying rising case numbers or unusual clusters of symptoms before formal confirmation. As a primary care scientist explained:

*I think it has tremendous potential to… policymakers… public health authorities aware of what’s happened on the ground… (if) we have noticed … cluster of patients who are showing up with … not part of the usual respiratory group of symptoms, and they are all associated with this mutation… this variant, so heads up*

[P05, Primary care scientist]

Second, some participants described EWS as supporting the screening and triage process by helping identify individuals at higher risks early in the care pathway, particularly in EDs. This was often discussed in relation to flagging symptoms or recent travel history associated with affected areas, enabling timely isolation and infection control measures. As an ED physician explained,

*Screen specifically for symptoms and travel exposures at triage in the Emergency Department… usually have a system… to isolate individuals who might be coming from affected areas… where new and emerging diseases have been flagged, it then gets flagged at triage, and then to help tell us whether people have to be isolated and then we very quickly get… the Infection Prevention and Control involved.*

P07, ED physician]

Finally, some participants described EWS as supporting informed clinical decision-making and outbreak preparedness by aggregating data across sources and helping anticipate which infections may be circulating in the community. As one infectious disease physician explained,

*We’re watching the respiratory tract infection epidemiology so we can predict what may be presenting in the clinic based on what is circulating in the community… We are in a pertussis epidemic in [province name]… cases are higher than baseline and when patients present with a cough, we consider that they have pertussis based on the epidemiology. So, this is highly relevant to my clinical practice.*

[P01, Infectious Disease physician]

### Theme 2: Desired Attributes of EWS for ARI

Participants identified 4 key attributes essential for an effective EWS for ARI, including accuracy, timeliness, integration, and equity. These qualities were viewed as critical for ensuring that the system reliably detects outbreaks, provides timely alerts, seamlessly integrates into clinical workflows, and addresses disparities in health care access.

#### Accuracy

Most participants emphasized that an effective EWS must balance sensitivity and specificity, often describing this balance in practical terms. Participants highlighted the importance of detecting “clear signals” of emerging outbreaks early (P02, ED physician), while avoiding frequent false alarms that could overwhelm clinicians or reduce confidence in the system. Concerns were raised that over-reliance on an inaccurate or overly sensitive system could erode trust and limit its usefulness in clinical practice.

*Doesn’t shoot out red flags constantly leading to alarm fatigue, but it also needs to be calibrated so that real early warnings that need to be warned about get the appropriate thought and reaction from policymakers and the public.*

P04, Infectious disease and critical care physician specialist]

To enhance accuracy, participants favored a multifactorial approach rather than reliance on a single metric, such as swab testing. A more comprehensive surveillance system was suggested to reduce biases and improve reliability.

*would have a multi-factorial kind of approach and wouldn’t rest on a single metric. Because these things are complex, the health system … and people…and communities…, I don’t think there is a silver bullet warning system like only swabs. Because if you pick one thing, it may have blind spots or biases baked into that particular approach. I would advocate a more holistic multi-factorial kind of approach for surveillance to recognize the complexity of the challenge of the problem we’re trying to solve.*

[P10, Family physician]

#### Timeliness

A key expectation was that an EWS should detect potential outbreaks in “Real-time” (P02, eED physician) and alert health care providers “within 24 to 72 hours” (P06, family physician). The ability to rapidly disseminate information was seen as critical for early clinical and public health interventions:

*Timeliness… that it could impact clinical care at the frontline*

[P08, Family physician]

*It would be timely in terms of getting the information out to the providers. And I would think within…short days. Ideally 48, 72 hours… would be helpful especially with respiratory illnesses that have potential to impact morbidity and mortality and impact kind of the need for healthcare providers that are looking after patients. So, timeliness would be the biggest one probably from the surveillance system perspective.*

[P08, Family physician]

#### Integration

Seamless integration into existing clinical systems was highlighted as a fundamental requirement. Participants stressed that the EWS should draw from multiple data sources, including electronic health records, swab testing data, wastewater surveillance, and self-reported symptoms, while minimizing disruptions to workflow.

*It’s ideally integrated into the electronic health records, so they only have to log into one system… I think a few things are ideal… everything from social media, wastewater, slight patterns, prescription medication use, symptoms that are recorded in health records and also people’s own self-reporting.*

[P06, Family physician]

#### Equity

Equity emerged as a core and widely emphasized attribute of an effective EWS across participants. Participants emphasized that an effective EWS must prioritize equity, ensuring equal access to testing and surveillance across urban and rural areas and marginalized populations. Key concerns included representation of “Indigenous and newcomer communities” (P03, Family physician), “equitable geographic coverage” (P04, Infectious disease and critical care physician specialist), and strict data privacy safeguards to prevent misuse.

*It would be really important that particular vulnerable groups that have been marginalised by the healthcare system over the last few decades were incorporated into the decision making around this so that an early warning system doesn’t further stigmatise them and that they would be wanting to participate. And… they could provide guidance on how it would need to be designed and implemented. So…our Indigenous communities… the example of having these separate TB clinics that did create a lot of inequities in the past. So, just being thoughtful about that piece. Because then we risk developing something that doesn’t actually help support the individuals that may be greatest risk for contracting respiratory illnesses.*

[P11, Family Physician]

Across practice contexts, participants consistently identified accuracy, timeliness, integration, and equity as core attributes of an effective EWS.

### Theme 3: Anticipated Challenges in EWS Implementation

Participants identified challenges at 4 key stages of EWS development, including initiation, implementation, use, and evaluation. While multiple concerns were raised for each stage, only the most predominant issues are reported below.

#### Initiation

Three primary challenges were highlighted regarding the initiation of an EWS for ARI: (1) lack of long-term commitment and high costs, (2) complexity of ARI mutations, and (3) public resistance due to data privacy concerns.

The lack of sustained commitment and financial investment was a major concern. Participants noted that enthusiasm and perhaps funding for pandemic-related technologies peaks during crises but diminishes between pandemics.

*One of the main lessons learned in the pandemic is everyone is interested during the pandemic, but no one is interested in the pandemic between the pandemics…you get all kinds of government funding and interest and global engagement when there is the threat but when there is no threat nobody wants to fund pandemic technology.*

[P01, Infectious disease physician]

The complexity of ARI mutations also raised concerns about the system’s practical utility. Participants questioned how an EWS could detect novel pathogens that had never been identified before.

*Now, there is new technology that might help us with PCR, and gene sequencing, and maybe we can hope to detect viruses that are previously unidentified more easily, but that stuff is really difficult… to then think about translating that at a clinical level, I mean, I don't know what to say about that… you can't detect anything that hasn’t been detected before.*

[P02, ED physician]

Finally, public trust and concerns about data misuse were seen as potential barriers to initiating an EWS.

*The governance of these types of systems, because… we’ve seen lots of concerns about corporations or others sucking up this type of data. So, it would need to be governed in a way that actually serves the people and the public and is not just generating profit.*

[P06, Family physician]

#### Implementation

Challenges related to data system incompatibility and lack of legislation on data sharing were emphasized as major barriers to implementation.

Participants described fragmentation across electronic health records and data systems, with different provinces and organizations using incompatible platforms.

*Different provinces have different electronic medical records. And a system would have to synchronize data from a variety of different sources so if it required a lot of tuning to the local electronic medical record, that would delay implementation.*

[P01, Infectious disease physician]

In addition, the absence of clear legislation on data sharing was noted as a challenge. The lack of standardized protocols for tracking and tracing infections during COVID-19 was cited as an example of system limitations.

*The contact tracing was non-existent in Ontario for the most part. It failed dramatically …restaurant sign up…(were) not be used by anyone. So, when people would test positive, there was no way to then see where they’ve been or anything… being able to contact trace better, that probably requires some sort of work from legislations at the legislation level.*

[P02, ED physician]

#### Use

Challenges related to workforce shortages, system capacity, and public engagement were the most frequently discussed barriers to EWS functionality. Almost all participants explicitly expressed their concerns related to the “administrative and workload burden” (10) associated with having a new EWS, especially given the chronic “human workforce shortage” (11). In one Canadian province, *“*2.5 million residents don’t have a primary care doctor” (11), and many rely on EDs, which are *“*typically really busy” (09). A participant stressed that,

*It’s hard for folks (in the ED) to add something else onto their busy list. They see so many people with very vague symptoms, it can be difficult to I think nail that down within say the triage or the first encounter with the patient.*

[09, Critical care physician]

Concerns were also raised about Canada’s ability to sustain robust testing and surveillance.

*If you have an atypical presentation and people do not have RSV* (respiratory syncytial virus)*, flu, or COVID, then that might lead you down the road of trying to investigate and discover that we’re dealing with something new. So, my worry is that our systems currently are not robust in terms of testing and isolating people with respiratory illness for the things that are circulating.*

[P07, ED physician]

Participants also noted that public trust and willingness to engage would influence the success of an EWS.

*We don’t have the greatest track record of using data in that equitable manner… so there is going to be an inherent amount of distress of the system because of our inability to really respond to data with high quality equitable interventions.*

[04, Infectious disease and critical care physician specialist]

Additionally, screening for travel history was seen as a critical function of EWS but also a potential source of discrimination.

*Everyone from Senegal is isolated for Ebola even though they’re presenting with they hurt their leg or something. I’m exaggerating, but so it can lead to a sense of discrimination when we’re making this based on where people have travelled from.*

[07, ED physician]

#### Evaluation

Discussions on evaluation focused less on challenges and more on suggested methods for assessing EWS effectiveness. Participants proposed that evaluation should consider data synchronization across sources, ease of use, and sensitivity and specificity of screening.

A participant envisioned an easy-to-use EWS that *“communicates with the EMR”* that *“flags”* warning as they *“input data”*, so they can perform *“point-of-care testing”*.

[P11, Family physician]

Another participant suggested evaluation through *“simulations and screening at airports… the evaluation happens frequently by people trying to get through and seeing if you pick that up through simulated events.”*

[P09, Critical care physician]

## Discussion

### Principal Findings

This qualitative study explored the perspectives of frontline clinicians and researchers involved in pandemic response on the role of EWS in monitoring and managing ARI in Canada. Three key themes emerged, including general knowledge of EWS, desired attributes, and anticipated challenges. While participants recognized the potential benefits of EWS, the absence of a widely recognized and established EWS for ARI in Canada limited their ability to engage in in-depth discussions about specific platforms. Participants identified accuracy, timeliness, integration, and equity as essential attributes of an effective system and highlighted challenges at each stage of development, including initiation, implementation, use, and evaluation. These findings underscore the complexities of designing and implementing a robust, sustainable, and equitable EWS for ARI in Canada. Including participants from diverse specialties and practice settings strengthened the relevance of our findings across varied clinical and health system contexts.

Our findings align with existing literature emphasizing that EWS primarily serves to detect outbreaks, guide clinical decision-making, and inform public health responses [36,37]. EWS has been shown to support real-time tracking of disease trends, early identification of emerging risks, and allocation of health care resources [38]. Similar to our participants’ perspectives, previous reviews highlight that effective EWS must be timely, accurate, and seamlessly integrated into existing health care workflows to maximize their utility [39]. Participants’ references to a 24‐72 hour alert window reflect the need for timely situational awareness to support public health and clinical response, rather than a specific evidence-based mortality threshold. Previous studies further suggest that “accuracy” in EWS reflects trade-offs between sensitivity and specificity. For example, increasing sensitivity can reduce missed events but often results in more false-positive alerts and greater alert burden, whereas prioritizing specificity may lower alert frequency but increase the risk of delayed detection of deterioration [39]. Participants’ concerns about alert fatigue and missed signals are consistent with these documented trade-offs.

In Canada, multiple surveillance systems monitor respiratory infections, including FluWatchers [17], TARRANET [18], and SPSN [19]. However, these systems primarily function as population-level surveillance tools and are not designed to provide real-time clinical decision support at the point of care. In contrast, several countries, including Australia, China, and parts of Europe, operate more integrated national respiratory infection surveillance systems that combine data from multiple sources, such as laboratory, syndromic, and sentinel surveillance [40,41]. In addition, some international EWS frameworks have begun integrating machine learning and artificial intelligence approaches to enhance early signal detection, identify high-risk populations, and support timely public health alerts [42,43]. Potential reasons for the disparity in EWS for respiratory infection between Canada and other countries may reflect a combination of system-level factors. First, Canada’s health system is highly decentralized, with responsibility for health care delivery and public health surveillance largely residing at the provincial and territorial levels. This governance structure can present challenges for the development of a unified, nationally integrated EWS. Second, ongoing variation in data infrastructure, as well as differences in privacy legislation and data-sharing agreements across jurisdictions, may limit the timely integration of surveillance data at a national level. Finally, persistent gaps in access to primary care in Canada, including a substantial proportion of individuals who are unattached to a regular primary care provider, may further constrain surveillance capacity. Individuals experiencing socioeconomic marginalization are disproportionately represented among this population, which may contribute to gaps in population-level monitoring. These findings point to the need for implementation-focused research to design and evaluate integrated EWS that link surveillance, clinical care, and public health decision-making in real time while minimizing alert fatigue and inequitable coverage.

### Challenges in EWS Development and Implementation

Participants identified challenges across all 4 stages of EWS development (initiation, implementation, use, and evaluation), with challenges at the initiation stage emerging as the most pressing concern. In particular, participants emphasized the lack of sustained investment and policy commitment to pandemic preparedness outside of crisis periods. While funding and interest peak during outbreaks, maintaining long-term support for EWS was viewed as difficult, a concern that aligns with prior research describing intermittent funding in surveillance infrastructure [44]. Participants noted that challenges during the initiation phase, such as limited sustained investment and unclear long-term priorities, often shaped downstream issues related to implementation, system fit, and long-term use.

Challenges related to implementation and use were also frequently discussed. Participants highlighted the fragmentation of electronic health records across provinces and the absence of standardized data-sharing protocols as major barriers to integrating EWS into clinical workflows. These issues are consistent with broader challenges observed in decentralized health care systems, where data silos and variable policies limit timely information exchange and generate real-time decision support [45]. In addition, workforce shortages, increased administrative burden, and limited system capacity were seen as factors that could further impede the adoption and effective use of EWS, reflecting system pressures that have been widely documented in the Canadian health context [46]. Prior studies of digital surveillance and decision-support implementation have shown that workforce shortages and competing clinical demands can limit engagement with alerting systems and reduce their effective use [39].

Public trust and engagement were identified as critical to the effectiveness of EWS, particularly in relation to data governance and equity. Participants emphasized concern that poorly designed or inadequately governed systems could exacerbate existing health disparities or undermine public confidence. Similar concerns have been noted in the digital health literature, where issues related to data validity, transparency, and legal framework have been shown to influence the utility and acceptability of surveillance technologies [47]. Prior Canadian and international studies have identified equity-related challenges in EWS implementation, including limited applicability to Indigenous, rural, and remote contexts and concerns around data governance and trust [48]. Participants raised similar issues, emphasizing Indigenous leadership, community-specific needs, and contextual relevance. To address these concerns, participants advocated for the importance of co-designing EWS with communities, particularly Indigenous and marginalized populations. Participants described co-design as involving early engagement with community leadership, shared decision-making around data governance and system priorities, and ongoing feedback during pilot testing and implementation. Evidence from disaster management and health equity suggests that co-design approaches can enhance cultural responsiveness and system relevance, supporting more equitable and effective implementation of digital surveillance tools [49,50].

### Opportunities and Future Directions

Despite these challenges, there is growing global momentum toward strengthening respiratory surveillance. Many countries have adopted disease-specific and syndromic surveillance models that rely on real-time data collection and clinical case reporting [51]. For example, the RESP-NET in the United States and Canada’s FluWatchers program demonstrates the feasibility of large-scale respiratory surveillance [52,53]. However, our findings suggest that existing surveillance efforts in Canada are not yet configured as a unified system that can support both public health monitoring and real-time frontline clinical decision-making. Moving forward, efforts should focus on designing an EWS that bridges these domains. Key considerations include improving interoperability between electronic health records, laboratory networks, and syndromic surveillance tools; establishing clear and consistent frameworks for data governance, privacy, and equitable access; and involving key stakeholders in system design to support cultural safety and equity in implementation.

In the Canadian context, advancing transparent governance and equitable data use will require clearer coordination across federal, provincial, and territorial jurisdictions. Participants’ emphasis on trust also points to the importance of embedding equity considerations early in system design, including meaningful engagement with communities that experience greater social and structural barriers. Together, these considerations may help enable Canada’s future EWS to be perceived as legitimate, trustworthy, and responsible to the needs of its diverse population.

### Limitations

This study has several limitations. First, we did not include policymakers, public health officials, and data scientists involved in the design and governance of EWS, whose perspectives may have provided additional insights for policy development, data integration strategies, and long-term sustainability. Second, interviews were conducted between August and October 2024, outside peak winter respiratory virus season. Participants’ perspectives may therefore reflect experiences during relatively stable surveillance periods rather than peak seasonal demand; however, discussions frequently drew on pandemic-era and outbreak experiences. Finally, patient perspectives were not included, limiting our understanding of public engagement and trust in EWS adoption.

### Conclusion

This study explored health care providers’ and researchers’ perspectives on EWS for ARI in Canada. Participants highlighted the potential of EWS to support outbreak detection, clinical decision-making, and pandemic preparedness, while also identifying persistent system-level challenges related to data integration, governance, and trust. These findings underscore the need for accurate, timely, integrated, and equitable systems that meaningfully bridge public health surveillance and clinical decision-making. Future research should incorporate policymaker and patient perspectives and examine how emerging digital and AI-enabled approaches can be responsibly integrated into EWS. With sustained investment and inclusive system design, Canada has the opportunity to strengthen its EWS capacity and improve preparedness for future respiratory outbreaks.

## Supplementary material

10.2196/85244 Multimedia Appendix 1 Semistructured interview guide.

10.2196/85244 Multimedia Appendix 2 Hierarchical coding structure.

10.2196/85244 Checklist 1 COREQ (Consolidated Criteria for Reporting Qualitative Research) checklist.

## Abbreviations

ARI: Acute Respiratory Infection
CIRN SOS: Canadian Immunization Research Network Serious Outcomes Surveillance
COREQ: Consolidated Criteria for Reporting Qualitative Research
ED: emergency department
EWS: early warning systems
HCP: health care provider
NEWS: National Early Warning Score
NEWS2: National Early Warning Score 2
RESP-NET: Respiratory Virus Hospitalization Surveillance Network
RSV: Respiratory Syncytial Virus
SPSN: Sentinel Practitioner Surveillance Network

## References

[1] Chen C, You Y, Du Y (2024). Global epidemiological trends in the incidence and deaths of acute respiratory infections from 1990 to 2021. Heliyon.

[2] Vos T, Lim SS, Abbafati C (2020). Global burden of 369 diseases and injuries in 204 countries and territories, 1990–2019: a systematic analysis for the Global Burden of Disease Study 2019. The Lancet.

[3] Kaye AD, Okeagu CN, Pham AD (2021). Economic impact of COVID-19 pandemic on healthcare facilities and systems: International perspectives. Best Pract Res Clin Anaesthesiol.

[4] Faramarzi A, Norouzi S, Dehdarirad H, Aghlmand S, Yusefzadeh H, Javan-Noughabi J (2024). The global economic burden of COVID-19 disease: a comprehensive systematic review and meta-analysis. Syst Rev.

[5] Shi T, Denouel A, Tietjen AK (2020). Global disease burden estimates of respiratory syncytial virus-associated acute respiratory infection in older adults in 2015: a systematic review and meta-analysis. J Infect Dis.

[6] (2021). Global health estimates: life expectancy and leading causes of death and disability. World Health Organization.

[7] Wood C, Chaboyer W, Carr P (2019). How do nurses use early warning scoring systems to detect and act on patient deterioration to ensure patient safety? A scoping review. Int J Nurs Stud.

[8] Roland D, Stilwell PA, Fortune PM, Alexander J, Clark SJ, Kenny S (2021). Case for change: a standardised inpatient paediatric early warning system in England. Arch Dis Child.

[9] Flenady T, Dwyer T, Sobolewska A (2020). Developing a sociocultural framework of compliance: an exploration of factors related to the use of early warning systems among acute care clinicians. BMC Health Serv Res.

[10] Graetz D, Kaye EC, Garza M (2020). Qualitative study of pediatric early warning systems’ impact on interdisciplinary communication in two pediatric oncology hospitals with varying resources. JCO Glob Oncol.

[11] McGaughey J, Fergusson DA, Van Bogaert P, Rose L (2021). Early warning systems and rapid response systems for the prevention of patient deterioration on acute adult hospital wards. Cochrane Database Syst Rev.

[12] Brangan E, Banks J, Brant H, Pullyblank A, Le Roux H, Redwood S (2018). Using the National Early Warning Score (NEWS) outside acute hospital settings: a qualitative study of staff experiences in the West of England. BMJ Open.

[13] Hoffmann M, Stengel S, Szecsenyi J, Peters-Klimm F (2022). Health care professionals’ perspectives on the utilisation of a remote surveillance and care tool for patients with COVID-19 in general practice: a qualitative study. BMC Prim Care.

[14] Morgan OW, Aguilera X, Ammon A (2021). Disease surveillance for the COVID-19 era: time for bold changes. The Lancet.

[15] Martin D, Razak F, Bayoumi I (2025). Primary care in the COVID-19 pandemic and beyond: lessons from Ontario. Can Fam Physician.

[16] Rowe BH, McRae A, Rosychuk RJ (2020). Temporal trends in emergency department volumes and crowding metrics in a western Canadian province: a population-based, administrative data study. BMC Health Serv Res.

[17] (2023). Government of Canada Flu (influenza): symptoms and treatment.

[18] University of Calgary University of calgary TARRANT viral watch. Welcome to TARRANT Viral Watch.

[19] (2019). Sentinel practitioner surveillance network (SPSN) — influenza vaccine effectiveness program. Public Health Ontario.

[20] McNeil S, Andrew M (2013). Serious outcomes surveillance (SOS) network. Canadian Immunization Research Network (CIRN).

[21] Kostakis I, Smith GB, Prytherch D (2021). The performance of the National Early Warning Score and National Early Warning Score 2 in hospitalised patients infected by the severe acute respiratory syndrome coronavirus 2 (SARS-CoV-2). Resuscitation.

[22] (2025). Respiratory virus hospitalization surveillance network (RESP-NET). Centers for disease control and prevention.

[23] Lee L, Desroches M, Mukhi S, Bancej C (2021). FluWatchers: evaluation of a crowdsourced influenza-like illness surveillance application for Canadian influenza seasons 2015-2016 to 2018-2019. Can Commun Dis Rep.

[24] McElroy T, Swartz EN, Hassani K (2019). Implementation study of a 5-component pediatric early warning system (PEWS) in an emergency department in British Columbia, Canada, to inform provincial scale up. BMC Emerg Med.

[25] Petersen JA, Rasmussen LS, Rydahl-Hansen S (2017). Barriers and facilitating factors related to use of early warning score among acute care nurses: a qualitative study. BMC Emerg Med.

[26] Tong A, Sainsbury P, Craig J (2007). Consolidated criteria for reporting qualitative research (COREQ): a 32-item checklist for interviews and focus groups. Int J Qual Health Care.

[27] Bearman M (2019). Focus on methodology: eliciting rich data: a practical approach to writing semi-structured interview schedules. FoHPE.

[28] DeJonckheere M, Vaughn LM (2019). Semistructured interviewing in primary care research: a balance of relationship and rigour. Fam Med Community Health.

[29] Braun V, Clarke V (2006). Using thematic analysis in psychology. Qual Res Psychol.

[30] Braun V, Clarke V (2012). APA Handbook of Research Methods in Psychology, Vol 2: Research Designs: Quantitative, Qualitative, Neuropsychological, and Biological.

[31] Braun V, Clarke V (2022). Conceptual and design thinking for thematic analysis. Qualitative Psychology.

[32] Graneheim UH, Lundman B (2004). Qualitative content analysis in nursing research: concepts, procedures and measures to achieve trustworthiness. Nurse Educ Today.

[33] Lindgren BM, Lundman B, Graneheim UH (2020). Abstraction and interpretation during the qualitative content analysis process. Int J Nurs Stud.

[34] Jamieson MK, Govaart GH, Pownall M (2023). Reflexivity in quantitative research: a rationale and beginner’s guide. Social and Personality Psych.

[35] Fusch P, Ness L (2015). Are we there yet? Data saturation in qualitative research. Qual Rep.

[36] Soucie JM (2012). Public health surveillance and data collection: general principles and impact on hemophilia care. Hematology.

[37] German RR, Lee LM, Horan JM (2001). Updated guidelines for evaluating public health surveillance systems: recommendations from the Guidelines Working Group. MMWR Recomm Rep.

[38] Patel D, Ayesha IE, Monson NR (2023). The effectiveness of metformin in diabetes prevention: a systematic review and meta-analysis. Cureus.

[39] Meckawy R, Stuckler D, Mehta A, Al-Ahdal T, Doebbeling BN (2022). Effectiveness of early warning systems in the detection of infectious diseases outbreaks: a systematic review. BMC Public Health.

[40] de Fougerolles TR, Damm O, Ansaldi F (2022). National influenza surveillance systems in five European countries: a qualitative comparative framework based on WHO guidance. BMC Public Health.

[41] El Guerche-Séblain C, Rigoine De Fougerolles T, Sampson K (2021). Comparison of influenza surveillance systems in Australia, China, Malaysia and expert recommendations for influenza control. BMC Public Health.

[42] Gao Y, Cai GY, Fang W (2020). Machine learning based early warning system enables accurate mortality risk prediction for COVID-19. Nat Commun.

[43] El Morr C, Ozdemir D, Asdaah Y, Saab A, El-Lahib Y, Sokhn ES (2024). AI-based epidemic and pandemic early warning systems: a systematic scoping review. Health Informatics J.

[44] Yang L, Weston C, Cude C, Kincl L (2020). Evaluating Oregon’s occupational public health surveillance system based on the CDC updated guidelines. Am J Ind Med.

[45] Quigley L, Lacombe-Duncan A, Adams S, Hepburn CM, Cohen E (2014). A qualitative analysis of information sharing for children with medical complexity within and across health care organizations. BMC Health Serv Res.

[46] Baumann A, Crea-Arsenio M (2023). The crisis in the nursing labour market: Canadian policy perspectives. Healthcare (Basel).

[47] Donelle L, Comer L, Hiebert B (2023). Use of digital technologies for public health surveillance during the COVID-19 pandemic: a scoping review. Digit Health.

[48] Govorchin A, Leduc M, Atleo CG, Hoogeveen D, Borgos I, Patrick L (2026). The right to health: indigenous data sovereignty in Canada during and beyond the COVID-19 pandemic. Lancet Reg Health Am.

[49] Tsai CH, Huang C, Chen YC, Zendejas E, Krafka S, Zendejas J (2024). Co-design smart disaster management systems with indigenous communities. Digit Gov: Res Pract.

[50] Gerrard J, Godwin S, Whiteley K, Charles J, Sadler S, Chuter V (2025). Co-design in healthcare with and for First Nations Peoples of the land now known as Australia: a narrative review. Int J Equity Health.

[51] Abat C, Chaudet H, Rolain JM, Colson P, Raoult D (2016). Traditional and syndromic surveillance of infectious diseases and pathogens. Int J Infect Dis.

[52] (2024). Respiratory virus hospitalization surveillance network (RESP-NET). Centers for Disease Control and Prevention (CDC).

[53] Ben Moussa M, Rahal A, Lee A, Mukhi S (2023). Syndromic surveillance performance in Canada throughout the COVID-19 pandemic, March 1, 2020 to March 4, 2023. Can Commun Dis Rep.

