# Supplementary material for: Health Care Providers’ Perspectives on Early Warning Systems for Acute Respiratory Infections in Canada: Qualitative Study
Source: JMIR Public Health Surveill. 2026 Apr 23;12:e85244. doi: 10.2196/85244 (PMC13105425; doi:10.2196/85244)
Supplement: Multimedia Appendix 1 [file publichealth-v12-e85244-s001.docx]

**Multimedia Appendix 2: Semi-structured Interview Guide**

**Pre-amble**

Hello *[name of participant],* my name is *[name of interviewer]* and I am a *[title]* with the Upstream Lab at St. Michael’s Hospital in Toronto. Thank you very much for taking part in this interview and taking the time to speak with me today. This interview will be about understanding your thoughts about the development of an early warning system for respiratory infections.

**Consent**

*Go through Oral Informed Consent Tool*

Do you have any questions at this point? *[Address any concerns; if none, continue]*

This interview should take around 45 minutes to an hour to complete and will be audio-recorded. It will be helpful for us if you speak slowly and clearly so that we can accurately transcribe this recording. Once you are ready, I will start the recording. *[Click ‘Record’ button]*

**Overview of Early Warning Systems**

I will first begin by asking some broad questions about Early Warning Systems (EWS). EWSs can reduce the impact of global infectious diseases by facilitating information exchange, monitoring, and early detection.

1. How would you describe your general knowledge of early warning systems?
2. Do you have any experience with early morning systems? If yes, which one?
3. What is your level of ease with adopting early warning system technology in your practice or healthcare setting?
4. Do you have any concerns regarding the use of an early warning surveillance system?
5. Do you have any expectations or needs that you would like to see with respect to an early warning system for respiratory infections?

**Thinking about Early Warning Systems data**

Next, I’ll ask you about your thoughts on an early warning surveillance system with respect to healthcare and managing patient needs.

1. Which ethical considerations should be addressed during the design and planning of an early warning system?
2. Can you identify which physiological parameters should be included in the early warning system for respiratory infections?
3. What do you think an ideal early warning surveillance system for respiratory infections would be like?
4. What are some limitations to relying on early warning systems for respiratory infections in the ED?
5. How long do you think it will take to develop and implement an early warning system for respiratory infections?
6. What are some considerations/lessons learned from the COVID-19 pandemic that can assist in the development and implementation of an early warning system for respiratory infections?

**Evaluating an Early Warning System**

For the last section, I will ask questions about how you might use EWSs in your practice to determine efficacy.

1. Can you think of any ways we might evaluate whether a new EWS is effective at identifying and alerting the medical team of respiratory infections?
2. Can you think of any factors that can influence the performance/accuracy of early warning systems to identify respiratory infections?
3. Can you identify any considerations about using an early warning system in your practice/organization that can influence performance or efficacy?
4. How would an early warning system to identify respiratory infections be incorporated into your current clinical workflow or organization?

Those are all the questions I have for you today. This concludes the interview. Thank you for your time; your participation is very much appreciated!
