# Supplementary material for: Health Care Providers’ Perspectives on Early Warning Systems for Acute Respiratory Infections in Canada: Qualitative Study
Source: JMIR Public Health Surveill. 2026 Apr 23;12:e85244. doi: 10.2196/85244 (PMC13105425; doi:10.2196/85244)
Supplement: Multimedia Appendix 2 [file publichealth-v12-e85244-s002.docx]

**Multimedia Appendix 3: Hierarchical coding structure of themes, subthemes, and example codes**

| **Theme** | **Subtheme** | **Example Codes** |
| --- | --- | --- |
| Theme 1: General knowledge of EWS for ARI | General awareness | Familiarity with EWS; Awareness of EWS; Knowledge of EWS |
|  | Insights and Tools learned from the COVID-19 pandemic | Lessons from COVID-19; Wastewater surveillance; Public health surveillance updates |
|  | Role of EWS in ARI management | Outbreak detection; Laboratory confirmation; Triage support; Clinical decision-making |
| Theme 2: Desired attributes of EWS for ARI | Accuracy | Signal reliability; Sensitivity–specificity trade-offs; Surveillance validity |
|  | Timeliness | Alerts within 24–72 hours; Real-time signals |
|  | Integration | Seamless workflow integration; Additional administrative burden |
|  | Equity | Indigenous communities; Marginalized populations; Data equity and privacy |
| Theme 3: Anticipated challenges in EWS implementation | Initiation | Funding instability; Policy commitment |
|  | Implementation | Infrastructure constraints; Interoperability barriers; Jurisdictional fragmentation |
|  | Use | Operational data fragmentation; Training and capacity building |
|  | Evaluation | Limited evaluative data; Challenges in system assessment |
